# The case for primary prevention of obesity in the era of GLP-1 therapies

**DOI:** 10.1016/j.lanepe.2026.101679

**Published:** 2026-04-16

**Authors:** Søren Holm, Manuel Tena-Sempere, Dimitrios Koutsouris, George V. Dedoussis, Karl-Heinz Wagner, Sara Arranz, Philippe Froguel, Torsten Bohn, Paulo J. Oliveira, Jeroen Lakerveld, OBEClust, Aakruti Kaikini, Achim Kramer, Adam Selamnia, Adina Weinberger, Adrian Jauch, Adriana Fontes, Adriana Voicu, Adrianna Tryskuc, Afshan Malik, Agnieszka Kozioł-Kozakowska, Agostino Di Ciaula, Agustin Fernandez, Aikaterina Vasileiou, Ainara Cano, Ainhoa Ruano, Alain Massart, Alberto Norhona, Alejandra Loyola Leyva, Aleksandra Davydova, Aleksandra Luszczynska, Ales Wodecki, Alessia D'Andrea, Alex Bravo Serrano, Alexandra Karahaliou, Alexandra Tiganouria, Alexandre Chikalanov, Alexandros Papadopoulos, Alexia Barroso, Alexis Kyriacou, Alfred Wagtendonk, Alice Denis, Alice Maguolo, Alicia Sicardi, Alkyoni Glympi, Alvaro Obeso, Amaia Barrena, Amalia Gastaldelli, Amelia Sarroca, Amëlie Bonnefond, Amine Belfoul, Amy Hough, Ana Cláudia Guedes, Ana Marcela Zapata Castellon, Ana Ochando, Ana Realinho, Ana Rito, Ana Teixeira, Anabel Martinez, Anabela Marisa Azul, Anastasios Delopoulos, Anastasios Papamanolis, Anders Eriksson, Anders Juul, André Lázaro, André Seabra, Andrea Devecchi, Andrea Piano Mortari, Andreas Jespersen, Andreas Vezakis, Andreea Ciudin-Mihai, Andreia Amaro, Andreia Silva, Anestis Dougkas, Aneta Radaczyńska, Angelica Dessì, Angie Jackson-Morris, Anika Kronberger, Anita Morandi, Anna Banik, Anna Brieva-Toloza, Anna Ek, Anna Kornafel, Anna Lena Aufschnaiter, Annalisa Roberti, Anne-Simone Parent, Anne Niknejad, Anneleen Segers, Anneli Rost, Anneline Pinson, Annemarie Olsen, Annette Schürmann, Annica Doersam, Antoni Caimari, António Cruz, Antonio Noto, Apostolos Malatras, Arianna D'Ulizia, Aristides Machado-Rodrigues, Armando Raimundo, Artur Mazur, Astrid Günther, Astrid Kemperman, Athanasia Kyrkili, Athanasios Anastasiou, Athanasios Kakasis, Augustina Jankauskiene, Ausrine Pliauckiene, Belén Pastor-Villaescusa, Benjamin Küther, Benny Lo, Bert Maier, Bianca Fuchs-Neuhold, Biljana Meshkovska, Billy Langlet, Binisha H. Mishra, Birk Schütz, Boshuizen Hendriek, Boyko Doychinov, Bruna Moreira, Bruno Manadas, Carina Magalhães, Carla Faria, Carlos Penha Gonçalves, Carlos Simón, Carmen Sayon-Orea, Carminda Morais, Carolina Ortega-Azorin, Cassandra Omane, Catalina Cuparencu, Catarina Parente, Cathrine Winding, Cátia Barra, Célia Cabral, Charlotte Ehlers Thomsen, Charlotte Jacquinet, Charlotte Ling, Charly Bastiaansen, Chloé Glachet, Christina Aguilar Riera, Christina Barragan Mesa, Christina Höfler, Christina Patmiou, Christine Ellersdorfer, Christine Poitou, Christoph Stahl, Christos Diou, Christos Nikitas, Chrysa Episkopou, Ciro Avolio, Claire Cannet, Cláudia Pereira, Coen Dros, Concepcion Aguilera, Constantinos Deltas, Cristina Barragan Yebra, Cristina Bouzas, Cristina Gora, Cristina Padez, Cristina Piras, Cristina Razquin, Cristinel Gheorghiu, Danai Kyrkou, Danai Malti, Danai Rossiou, Daniela Grach, Daniela Rodrigues, Daniela Rosendo-Silva, Danika Shepis, Darya Silchenko, David Horner, David Thivel, Dawei Chen, Debora Porri, Delfien Gryspeerdt, Desiree Lucassen, Diana Juanita Mora, Diana Sousa, Dimitrios Aletras, Dimitrios Fotiadis, Dimitrios Koutsouris, Dimitrios Tsakalidis, Dimitrios Zaikis, Dimitris Gkoulis, Dimitris Plakas, Djamel Rahmani, Dolores Corella, Domenico Corica, Dominika Malińska, Dominiki Gallou, Dorota Drożdż, Dorota Komar, Dorret I. Boomsma, Edith Feskens, Eduard Mogas Vinals, Eduardo Aguaviva, Eduardo Lopes, Edyta Łuszczki, Egeria Scoditti, Eirini Bathrellou, Eirini Marouli, Eleftheria Vellidou, Elena Ferragut Roig, Elena Jansen, Elena Patra, Elena Santacruz, Eleni Chatzi, Eleni Georga, Eleni Politi, Eleni Ramouzi, Elisabeth Thiering, Ellena Badrick, Elsa Sousa De Lamy, Emiilie de Zoete, Empar Lurbe, Ena Nielsen, Eran Segal, Erica van den Akker, Ermelindo Leal, Eszter Salamon, Eudald Casals Mercadal, Eugénia Carvalho, Eva Karaglani, Eva Schernhammer, Eva Schernhammer, Eva Winzer, Evangelia Charmandari, Evgenia Lampropoulou, Evika Karamaggioli, Ezgi Kolay, Fabio Pfaehler, Fahmida Sarker, Farhad Vahid, Fátima Martins, Federica Pinto, Felip Vilella, Fernando Capela e Silva, Fernando Fernádez-Aranda, Fernando Veloso, Flávio Reis, Fleur Hukema, Florence Flamein, Florence Mehl, Florian Junne, Francesca Marazzi, Francesco Agnoloni, Francisco Jesús Llorente-Cantarero, Francisco Pereira, Franco Sassi, Frédéric Burdet, Frédéric Gottrand, Frediana Tummino, Fügen Çullu Çokuğraş, Gabin Drouard, Gary Frost, Gemma Vilahur, George-Mihael Manea, George Dedoussis, George Dimitrakopoulos, George Matsopoulos, George Mylonas, Georgios Saltaouras, Georgios Theodoridis, Gerard Marrugat, Gerhardus Ansgar, Giada Martello, Gianna Karanasiou, Giannis Arnaoutis, Gillian Santorelli, Giorgia Pepe, Gitte Ravn-Haren, Giuseppe Masanotti, Giuseppe Tarantino, Gloria Fackelmann, Graciela Gastelum Varela, Grzegorz Sumara, Guadalupe González, Guillaume Vanotti, Gunda Herberth, Gunopulos Dimitrios, Haluk Cezmi Çokuğraş, Hanna Zaleskiewicz, Hans Zischka, Harshitha Shanmugam, Hartmut Schäfer, Heidi Lammers-van der Holst, Heike Vogel, Helen Gika, Helena Nogueira, Helena Rodrigues, Helene Devroye, Helene Reinbach, Helmut Schröder, Heloísa Gerardo, Hoang-Ha Nguyen, Ieva Jura Paulaviciene, Ifigeneia Rizopoulou, Imke Matullat, Ines Barretxeguren, Inge Depoortere, Ingo Klarholz, Ioanna Panagiota Kalafati, Ioannis Ioakeimidis, Ioannis Kakkos, Ioannis Pagkalos, Ioannis Papathanail, Ioannis Sarafis, Ioannis Vezakis, Ioannis Vondikakis, Irina Carpusca, Iris Mangelschots, Isabel Araújo, Isabel Garcia Perez, Isabel Santonja, Isabelle Mack, Ismini Grapsa, Itziar Tueros, Iulian Dragan, Ivana Vaclavkova, Ivo van Delft, Izidor Mlakar, Jaakko Kaprio, Jack Olney, Jakob Tarp, Jakub Marecek, Jan Eriksson, Jan Janssen, Jana Selent, Jana Throm, Jaroslaw Rakoczy, Javier Amézaga, Javier Carrero, Javier Gonzalez, Javier Menéndez, Jayne Evans, Jeanne Lagerweij, Jennifer Kefauver, Jeroen Lakerveld, Jet van de Geest, Jihan Halimi, Jilani Hannah, Jingmin Zhu, Jo Boulding, Joan Teichenné, Joana Sacramento, Joanneum Austria, João Filipe Raposo, João Lima, João Ramalho-Santos, John Filippas, John Jones, John Wright, Joline Beulens, Jonathan Turner, Joost Wesseling, Joram Posma, Joreintje Mackenbach, Jorge Abrantes, Jorge Ribeiro, Jorrit van Uhm, Jose Raul Herance Camacho, José Teixeira, José Vicente Sorlí, Josean Montoya, Josep A. Tur, Josine Stuber, Jouko Miettunen, Juan José Alba, Juan Ramon Tejedor, Juan Roa, Julia Díez, Juliane Halftermeyer, Julie-Anne Nazare, Julie Dam, Julie Fudvoye, Julio Álvarez Pitti, Justė Parnarauskienė, Justiina Ronkainen, Justyna Wyszyńska, Kajus Merkevicius, Kalogeraki Vasiliki, Kamille Almer Bernsdorf Torp, Karl-Heinz Wagner, Karl Bacos, Karnaki Pania, Karolina Krystyna Kopeć, Karolina Czarnecka, Karolis Azukaitis, Karri Silventoinen, Katarzyna Binder-Olibrowsk, Katarzyna Janiszewska, Katherine Flores-Rojas, Katrin Giel, Katrin Ziser, Khin Hlaing, Kilian Gandolf, Kinga Zel-Hans, Kirsten Schroll Bjørnsbo, Klaus Bønnelykke, Konstantina Maria Togka, Konstantina Chachlaki, Kristian Almstrup, Kyriaki Papantoniou, Lars Dragsted, Laura García, Laura Herrera, Laura Mewes, Laurent Malisoux, Leah Lund, Leila Mathy, Lelita Santos, Leo-Pekka Lyytikäinen, Letteria Morabito, Liam Walsh, Licinio Manco, Liesbeth van Rossum, Lieven Annemans, Liliana Cori, Logan Stuck, Lorena Calderón Pérez, Lou Götz, Louise Seconda, Lubnaa Abdur Rahman, Lucero Munguia, Lucia Brodosi, Lucía Camacho, Lucrezia Bertoni, Luigi Atzori, Luigi Petito, Luis Cereijo, Luís Grilo, Luís Rama, Maartje van den Belt, Magdalena Wieczorkowska, Magdalena Wrzesinska, Magdalena Zebrowska, Mahesh Desai, Maira Bes-Rastrollo, Malgorzata Gabriela Wasniewska, Małgorzata Wójcik, Manon Gantenbein, Manuel Franco, Manuel Tena-Sempere, Marco Mensink, Margarida Liz, Maria Cristina Morelli, Maria Giovanna Onorati, María José de la Torre Aguilar, Maria Paula Macedo, Maria Raquel Silva, Maria Teresa Cruz, Maria-Dolores Sole, Maria Hassapidou, María Jesús Vázquez, Maria Kafyra, María Mercedes Gil-Campos, Maria Pereira, Maria Perez Jimenez, Maria Siwa, Maria Wakolbinger, Marialetizia Rastelli, Marianna Kalliostra, Marianna Panagiotidou, Marie Shrestha, Marie Standl, Marilena Tarousi, Marina Papadopoulou, Marina Rodenas Munar, Mario Fernandez Fraga, Mariusz Wieckowski, Mariya Zheleva, Mark Ibberson, Mark Kozdoba, Marlene Lages, Marlene Rechtsteiner, Marlies Wallner, Marta Comes Martinez, Marta Gaspar, Marthe Smedinga, Martin Bigec, Mary Giannakoulia, Matilde Vicente, Matt O' Flynn, Matteo Colombo, Matteo Mauri, Mavis Fosuaa Boateng, Meeke Ummels, Mercedes Caro, Meriem Ouni, Meropi Kontogianni, Michel Vaillant, Michele Stecchi, Miguel A. Martinez-Gonzalez, Miguel Adriano Sanchez-Lastra, Miguel Angel Sanchez-Garrido, Miguel Castelo-Branco, Miriam Ressler, Miriam Ulz, Mirosław Bik-Multaowski, Mohamad Khalil, Mohan Raju, Monica Truninger, Monika Kolska, Monika Riederer, Monique Vingerhoeds, Nadia Micali, Nanna Lien, Natalia Dąbek, Natalia Paduszyńska, Natalia Plociennik-Korycka, Natalia Zaldua, Nick Martin, Nick Verhaeghe, Nicola Segata, Nieves Embade, Nikolaus Forgó, Nikos Alimpertis, Nikos Sintoris, Nina Mononen, Nishit Patel, Noemi Rita Colacione, Norbert Schmitz, Nuno Batalha, Nuno Lourenço, Nuno Madeira, Núria Canela, Olga Begou, Olga Deda, Olga Glazunova, Olga Portolés, Olga Startseva, Ömer Faruk Beser, Orestis Papagiannopoulos, Orla O’Sullivan, Oscar Carrancio, Oscar Coltell, Oscar Millet, Pablo Santamarina, Panagiota Veloudi, Panagiotis Alimisis, Panagiotis Demestichas, Panagiotis Moulos, Panagiotis Symianakis, Paris Kantaras, Pashupati P. Mishra, Patrícia Afonso-Mendes, Patrícia Jakubek-Olszewska, Patrícia Vieira, Patrizia Baire, Patrizia Zitelli, Paul Cotter, Paul Hardman, Paulina Krzywicka, Paulo Matafome, Paulo Oliveira, Pavel Rytir, Pedro Faria, Pedro Ferreira, Pedro Santos, Pelin Alpay, Penio Kassari, Petar Atanasov, Peter Suenaert, Petr Rysavy, Petra Van Haren, Philippe Froguel, Piero Portincasa, Pierre-Yves Barelle, Pieter van Gorp, Pietro Dionisio, Pietro Monti, Polina Dobroslavska, Przemko Kwinta, Quentin Terwagne, Radka Savova, Rafael Pineda, Ralf Jockers, Ramon Que, Raquel Henriques, Raul Fernandez, Rebecca Kofod Vinding, Rebecca Markilie, Renate Meeusen, René Pool, Ricardo Conde, Richard Wynne, Rikke Andersen, Rita Oliveira, Rita Patarrão, Rita Pinheiro, Robert Shorten, Robertas Kemezys, Roberto Valiente, Robin Liechti, Rocio Urdinguio, Rodessa May Marquez, Rooholla Poursoleymani, Rosalie Bakker, Roumen Nikolov, Rozenn Nedelec, Rubén Gil, Ruben Willems, Rui Pimenta, Rui Tavares, Rutger Laterveer, Sabin Linaza, Salvador Fernández-Arroyo, Sandra Haider, Sanne Roove, Sara Amaral, Sara Arranz, Sara Casartelli, Sarah Forberger, Scott Gordon, Sebastien Bouret, Serap Erdine, Serena Rinaldi, Serge Autexier, Sergej Černčič, Shanti Neff-Baro, Shie Mannor, Sílvia Conde, Silvia Garcia, Silvia Sabatini, Simon Berner, Sofia-Maria Genitsaridi, Sofia Silvola, Sofia Tavares, Sondre H. Herstad, Sónia Pinho, Soren Holm, Sreekala Nampoothiri, Stavros Gravas, Stavros Milioulis, Stavros Pitoglou, Stavroula Mougiakakou, Stefanie Vandevijvere, Stephan Kampshoff, Stephan Zipfel, Stephane Lobbens, Steve Bell, Stine Agergaard Holmboe, Stuart McLennan, Subhechchha Bhandari, Susana Jiménez, Susann Weihrauch-Blüher, Susanna Pätsi, Susanne Hetty, Susanne Kröber, Suzan Evers, Suzanne Bruins, Svetlin Hansov, Sylvain Sebert, Tamara Schikowski, Tatiana Burrinha, Teemu Palviainen, Teresa Cunha-Oliveira, Terho Lehtimäki, Thao Minh Lam, Thelma Androutsou, Thibaut Galba, Tina Termansen, Tiziana De Magistris, Tommaso Aversa, Torsten Bohn, Trine Koch Hueg, Ulf Ekelund, Ulla Toft, Umberto Restrelli, Urska Smrke, Urte Klink, Valentin Kraus, Valeria Cuenca, Valerios Chatzianastasiou, Van Du Thoung Tran, Vanessa Bullon-Vela, Vanessa Schoissengeier, Vangelis Argoudelis, Vasiliki Bountziouka, Vasiliki Vavouraki, Vassiliki Moumtzi, Vassiliki Papageorgiou-Anagnostou, Vassilios Fanos, Vassilis Apostolakos, Ventsislav Nikolov, Vera Stavroulaki, Vilma Sardão, Vincent Prevot, Vincenzo Atella, Virginia Lopez, Wender Bredie, Willem de Vos, Xiaoyu He, Yannick Naudet, Yannis Manios, Yassine Talas, Ying Wang, Yioula Lekka, Yoanna Ivanova, Zana Antonova, Zein Kallas, Zofia Szczuka

**Affiliations:** a Centre for Social Ethics and Policy, School of Law, University of Manchester, Manchester M13 9PL, UK; b Center for Medical Ethics, Institute of Health and Society (HELSAM), University of Oslo, Oslo 0318, Norway; c Department of Cell Biology, Physiology and Immunology, University of Córdoba, Spain; d Biomedical Engineering Laboratory, Institute of Communication and Computer Systems (ICCS), Athens, 10682, Greece; e Department of Nutrition and Dietetics, School of Health Science and Education, Harokopio University of Athens, Athens, 17676, Greece; f Department of Nutritional Sciences, University of Vienna, Austria; g AZTI, Food Research, Basque Research and Technology Alliance (BRTA), Parque Tecnológico de Bizkaia, Astondo Bidea, Edificio 609, Derio - Bizkaia, 48160, Spain; h Université de Lille, Inserm UMR1283, CNRS UMR8199, European Genomic Institute for Diabetes (EGID), Institut Pasteur de Lille, University Hospital, Lille, France; i Nutrition and Health Research Group, Department of Precision Health, Luxembourg Institute of Health, L-1445 Strassen, Luxembourg; j CNC-UC, Center for Neuroscience and Cell Biology, University of Coimbra, Portugal; k CiBB, Center for Innovative Biomedicine and Biotechnology, University of Coimbra, Portugal; l Department of Epidemiology and Data Science, Amsterdam Public Health Research Institute, Amsterdam University Medical Center, Vrije Universiteit Amsterdam, Amsterdam, the Netherlands; m Instituto Maimónides de Investigación Biomédica de Córdoba (IMIBIC), and CIBER Fisiopatología de la Obesidad y Nutrición, Instituto de Salud Carlos III Córdoba, 14004, Spain; n Research Platform Active Aging, University of Vienna, Austria; o Department of Metabolism, Imperial College London, London, UK

**Keywords:** Obesity, Prevention, Healthcare costs, Weight gain, GLP-1 receptor agonists

## Abstract

Obesity rates continue to rise across Europe, driven by obesogenic environments shaped by food systems, urban design, socioeconomic inequalities, and commercial forces. The introduction of incretin-based pharmacotherapies like GLP-1 receptor agonists has transformed treatment, leading to significant weight loss and metabolic benefits for many. This progress has shifted public and policy discourse, with some suggesting that effective treatment reduces the urgency of prevention. However, this position paper argues that the growing availability of medications actually reinforces the need to prioritize primary prevention. While pharmacotherapy can improve individual outcomes, it cannot lower population-level risk or deliver the wider societal, environmental, and economic benefits of prevention. The paper also outlines key policy implications to reaffirm prevention as a cornerstone of public health across Europe.

## Introduction

Obesity is one of the defining health challenges of our time, affecting almost a quarter of all adults in the European region and contributing to diabetes, cardiovascular disease, several cancers, and reduced quality of life.^1^^,^^2^ Recognized as a chronic, relapsing disease in its own right,^3^^,^^4^ obesity is associated with numerous co-morbidities including musculoskeletal disorders and multiple cancers,^5^^,^^6^ and its socio-economic burden is enormous, accounting for up to 6–8% of total health-related costs when including productivity losses.^7^ Despite decades of awareness of its determinants, obesity prevalence continues to rise, driven by insufficient implementation of effective interventions, persistent structural factors such as unhealthy food and physical activity environments, and socio-economic inequalities. Against this backdrop, the emergence of long-acting incretin therapies–especially glucagon-like peptide 1 (GLP-1) receptor agonists–has transformed both clinical practice and the societal discourse on obesity.

**Key messages**

- The arrival of GLP-1 receptor agonists marks a major advance in obesity management but does not treat the underlying cause and thus does not reduce the need for strong primary prevention.
- Prevention and treatment are complementary, but not equal priorities: preventive action requires greater and more sustained investment to achieve population-wide and long-term benefits.
- Both prevention and treatment are needed and investment in one does not mean disinvestment in the other.
- Pharmacological treatment addresses individual outcomes but leaves the societal, commercial, and environmental drivers of obesity untouched.
- Strengthening policies and environments that make healthy choices easy remains essential for sustainable, equitable, and economically viable control of obesity in Europe and worldwide.

These drugs (including semaglutide and tirzepatide) have proven effective for short-term weight reduction and improvement of metabolic parameters in large subgroups.^8^ While long-term data are still emerging, current evidence suggests that sustained benefits depend on continued treatment.^9^ They are still, without question, a major therapeutic advance. However, their success has also prompted a significant shift in the political and public conversation. The promise of pharmacological solutions is already starting to divert attention and investment away from prevention, as if obesity were now primarily a clinical rather than a societal challenge. The argument that ‘we now have effective treatment, so prevention is less urgent’ is increasingly heard in policy circles, the (social) media, and the public. Even though this simplistic dichotomy is problematised and responded to in the academic literature and in reports from official bodies and think-tanks, it still influences the public discourse.^10^, ^11^, ^12^, ^13^ Despite the relief that GLP-1 drugs can bring to individuals, treatment is long-term and costly, and it does only treat the symptoms and some established individual drivers, not the primary causes of obesity.

This position paper argues the opposite: that the emergence of GLP-1 drugs strengthens, rather than weakens the case for prioritising primary prevention of obesity. The prevention of obesity remains essential because these pharmacological treatments, while effective for many individuals, do not address the fundamental drivers of the obesity epidemic and cannot deliver the broader health, social, economic, and environmental co-benefits associated with preventive action.

## Scope of the paper

This paper focuses on the continued importance of primary prevention of obesity in high-income countries, with a particular focus on Europe. It is not an argument against treatment or against equitable access to new anti-obesity medications. Nor does it discuss in detail whether GLP-1 drugs should be publicly reimbursed or how eligibility criteria should be defined.

While prevention and treatment may compete for political attention, they rarely draw from the same budgets.^14^^,^^15^ Reducing investment in prevention does not automatically result in increased resources for treatment. Even if both prevention and treatment are part of the same health budget, any savings from obesity prevention are not necessarily reallocated to obesity treatment or to other health or non-health spending areas. Rather than framing prevention and treatment as competing priorities, this paper emphasises their complementarity but also argues that prevention requires renewed strategic and financial commitment.

## The structural drivers of obesity

Despite decades of research and numerous interventions, obesity prevalence continues to rise in most European countries.^16^ These trends do not reflect a lack of willpower among individuals, as generally thought, but rather the structural and commercial forces that shape our environments and behaviours.^17^^,^^18^ The modern food system produces an abundance of inexpensive ultra-processed, energy-dense, nutrient-poor products, environmental and ecological degradation while marketing practices drive overconsumption; and urban design often discourages physical activity, encourages sedentary behaviours and disconnects with nature. These ‘obesogenic environments’ interact with genetic, biological, environmental, ecological, socio-economic, and psychosocial factors to sustain high and growing obesity rates, and associated diseases.^19^

## Treatment with GLP-1 agonists, a new era in treatment

GLP-1 receptor agonists modulate on hormonal pathways involved in appetite regulation and glucose metabolism, altering the hedonic valuation of food.^20^ In randomised controlled trials, drugs such as semaglutide (a pure GLP-1 receptor agonist) and tirzepatide (dual GLP-1 and GIP agonist) have demonstrated average weight losses of 10–20% of initial body weight over one to two years, along with improvements in glycaemic control, blood pressure, and lipid profiles.^21^

However, efficacy comes with caveats. Gastrointestinal side effects are common and often lead to discontinuation.^22^ Although safety has been evaluated in clinical trials, uncertainties remain about the longer-term developmental and behavioural consequences of sustained appetite suppression, particularly in children and adolescents. Marked reductions in energy intake may affect dietary quality, macronutrient balance, and lean mass accretion during critical growth periods, and the psychological effects of prolonged pharmacological appetite modulation in youth are not yet fully understood. In real-world use, discontinuation rates are substantial, and most individuals regain a large proportion of lost weight after stopping treatment.^22^

Importantly, these medications are expensive, and their cost-effectiveness depends heavily on long-term adherence and health system context. Although generic versions may reduce prices over time, experience with insulin analogues suggests that pharmaceutical innovation and marketing will maintain high costs for newer formulations. This has, for instance, happened with insulin,^23^ and the same companies, such as Novo Nordisk and Eli Lilly, dominate both markets.

## Why prevention remains essential

### Obesity is a societal challenge, not merely an individual problem

Pharmaceutical treatments target individuals that have developed clinical obesity, but the obesity epidemic is driven by collective, upstream determinants: food systems, urban planning, socioeconomic inequality, and commercial practices.^17^ Moreover, early biological determinants, linked to environmental factors and occurring during critical developmental windows, from gestation to infancy, contribute to obesity development at the population level.^19^^,^^24^ Overemphasis on pharmacological solutions risks reinforcing ‘individual responsibilisation’: the notion that people are solely responsible for their weight. This framing is ethically problematic and empirically flawed, and may be a particular risk in this context because obesity is already strongly socially stigmatised.^25^ Sustainable reductions in obesity prevalence require structural changes, not just individual medical management that may cure symptoms, not causes.

### Treatment does not remove the biological and psychological drivers of obesity

Even with the best available drugs, obesity remains difficult to treat durably. Biological adaptations to weight loss–including reductions in basal metabolic rate, increased appetite signalling, and changes in adipose tissue metabolism–promote weight regain.^26^ Evidence that obesity creates long-lasting epigenetic ‘memory’ that promotes weight regain highlights why early preventive interventions are likely more effective than later treatment.^27^ Psychological mechanisms such as hedonic eating and food cue reactivity are also resilient. Animal and human studies suggest that chronic overnutrition induces epigenetic changes that may persist across generations.^28^

In other words, while GLP-1 drugs can reset body weight temporarily, they do not reset the underlying causes, i.e., environment, lifestyles or biology that foster obesity. Without parallel preventive action, each new generation will face the same risks.

### The environment remains obesogenic

Pharmacotherapy does not alter the environments that promote excessive caloric intake within unbalanced diets and sedentary behaviour. Obesogenic exposures (including unhealthy food and marketing landscapes, car-oriented urban design, and limited opportunities for physical activity) operate alongside broader social, economic, commercial, and built-environmental conditions, as well as exposure to pollutants such as endocrine-disrupting chemicals, to shape behaviour, metabolic regulation, and obesity risk. These influences permeate daily life, from portion sizes and product placement to digital marketing and incentives that reinforce passive travel habits. As long as these drivers persist, treatment will remain reactive and perpetual rather than preventive and transformative.

### Prevention has multiple individual, societal, and environmental co-benefits

Policies that promote healthy diets, physical activity, and equitable access to supportive environments yield broad benefits extending far beyond obesity. They reduce cardiovascular disease, diabetes, and some types of cancer; improve mental well-being and productivity; and contribute to climate, environmental and biodiversity goals through more sustainable food systems and transport patterns.^29^ These co-benefits make such upstream primary and secondary prevention approaches a high-value investment even if obesity prevalence were not rising.

Offering combined lifestyle programmes as secondary prevention or as a management strategy (either alone or alongside other treatments) also provides multiple co-benefits. Continuing to provide these programmes is essential, particularly to support individuals in maintaining weight loss after drug therapy.

### Prevention supports treatment and maintenance

Preventive environments also aid those who have lost weight through pharmacological or behavioural means. They support healthy food purchases, active transport (walking and cycling) in an equitable way; crucial for long-term maintenance of a healthy body composition. As such, they effectively support turning primary prevention into secondary prevention. The other way around is also possible, that wide use of GLP-1 drugs could eventually reduce availability of unhealthy foods, through reduced demand, although GLP-1 drugs do not in themselves lead to improved nutritional quality.^30^

### Economic sustainability

The long-term affordability of GLP-1-based treatments at population scale is uncertain. Even with partial price reductions, widespread use would represent a substantial and recurring expenditure for health systems. As long as the systemic drivers of obesity at a population level are not addressed, more and more people will develop obesity, which will drive up the costs of treatment in the future. Prevention, in contrast, offers durable returns, and can be implemented also in less developed countries, which carry a large burden of the global obesity pandemia. Like insulin for diabetes, GLP-1 drugs are likely to remain valuable but costly chronic therapies, not one-time cures. Investing in pharmacological treatment without addressing root causes will create escalating costs without solving the underlying problem.

## Policy implications

An effective and balanced policy for addressing obesity should recognise the complementary roles of prevention and treatment. Pharmacotherapy expands the toolbox but must not replace structural approaches that target the root causes of overweight and obesity. Striking such a balance does not mean that both sides require equal investment; preventive approaches necessarily demand greater and more sustained commitment, given their broader population impact and long-term benefits. While structural prevention can face considerable political and practical barriers (including commercial resistance and debates over regulatory scope) real-world experience shows that measures such as sugar-sweetened beverage taxes, marketing restrictions to children, urban design measures promoting active transport, and product reformulation can produce measurable effects.^31^, ^32^, ^33^, ^34^ The barriers do not weaken the case for prevention; rather, they highlight the need for sustained political commitment and demand ambitious reform on many fronts and on a scale sufficient to address the sum of environmental influences that exacerbate the likelihood of obesity in individuals or populations and in different settings.^35^ See Panel 1 for a list of policy implications.

Panel 1 **Policy implications.**

European and national health policies should:

1. **Reaffirm prevention as the cornerstone** of obesity strategy in European and national public health frameworks.
2. **Invest in structural and regulatory interventions**, including restrictions on unhealthy food marketing, reformulation targets, implementation of objective front of pack labelling and fiscal measures to incentivise healthier choices.
3. **Strengthen environments** that support physical activity and active transport through urban, workplace and school design, financial and social structures that promote walking, cycling and public transport use.
4. **Address the barriers**—barriers for prevention should not be a reason to rely solely on treatment, but a reason to increase preventive efforts
5. **Integrate primary, secondary, and tertiary prevention and treatment**, ensuring that individuals who use GLP-1 drugs (or receive other treatments such as bariatric surgery) receive long-term behavioural and environmental support to sustain benefits.
6. **Monitor equity impacts**, as expensive pharmacotherapy risks widening socioeconomic inequalities in obesity and related diseases.
7. **Support research and innovation** in preventive systems approaches, including cross-sectoral co-created policies linking health, environment, and economy.

## Conclusion

The obesity epidemic remains one of Europe’s defining public health challenges and primary prevention should not only be at the heart of all strategies but receive even greater emphasis and investment to achieve sustainable and equitable health improvements. GLP-1 receptor agonists represent a remarkable scientific and therapeutic breakthrough, and they offer real hope for individuals living with obesity and should be accessible to those who need them. Yet, their promise does not diminish the urgency of preventing obesity in the first place, as obesity is driven by structural forces that no medication can neutralise.

Primary prevention–through healthier and sustainable food systems, active and safer environments, and equitable social policies–remains indispensable. Far from rendering prevention obsolete, the pharmacological revolution in obesity treatment highlights the limits of medical solutions and the enduring necessity of addressing the root causes of obesity. The continued prioritisation of prevention is essential not only for health, but for the sustainability and fairness of our societies.

## Contributors

The manuscript was conceived and initial drafts were prepared by SH and JL. MTS, DK, GVD, KHW, SA, PF, TB and PJO provided conceptual and textual input on the draft. A pre-final version was circulated among the OBEClust consortia which resulted in further revisions, which were checked and approved by all authors.

## Declaration of interests

SH: No conflicts of interest.

MTS: No conflicts of interest.

DK: No conflicts of interest.

GVD: No conflicts of interest.

KHW: No conflict of interest.

SA: No conflict of interest.

PhF: No conflict of interest.

TB: No conflict of interest.

PJO: Support received from the Foundation for Science and Technology, Portugal; MAPFRE Foundation European Union, and from Elsevier from book sales.

JL: No conflicts of interest.

## Appendix A Supplementary data

Annex 1-Wordfile

Collaborator list
