# Supplementary material for: The case for primary prevention of obesity in the era of GLP-1 therapies
Source: Lancet Reg Health Eur. 2026 Apr 16;66:101679. doi: 10.1016/j.lanepe.2026.101679 (PMC13330260; doi:10.1016/j.lanepe.2026.101679)
Supplement: Annex 1-Wordfile [file mmc1.docx]

Annex 1

OBEClust is the European cluster of nine large obesity research projects: OBCT; BETTER4U; PAS GRAS; Obelisk; BioStreams; eprObes; CoDiet; HealthyW8; and Shift2Health.

For each project the affiliated organisations and consortium members are listed below.^[[1]](#endnote-1)^

**OBCT** (Obesity: biological, sociocultural and environmental risk trajectories)

**Amsterdam UMC**

Jeroen Lakerveld

Thao Minh Lam

Joline Beulens

Joreintje Mackenbach

Jet van de Geest

Alfred Wagtendonk

Nishit Patel

Mohan Raju

Lucrezia Bertoni

Josine Stuber

Hoang-Ha Nguyen

Rosalie Bakker

**Erasmus Medical Center**

Liesbeth van Rossum

Erica van den Akker

Renate Meeusen

Jorrit van Uhm

**Imperial College of Science Technology and Medicine**

Franco Sassi

Jingmin Zhu

**Norwegian School of Sport Sciences**

Ulf Ekelund

Miguel Adriano Sanchez-Lastra

Jakob Tarp

**Region Hovestaden - Frederiksberg Hospital**

Ulla Toft

Kamille Almer Bernsdorf Torp

Kirsten Schroll Bjørnsbo

Tina Termansen

**SWPS University**

Aleksandra Luszczynska

Anna Banik

Maria Siwa

Natalia Paduszyńska

**Universidad de Alcalá**

Julia Díez

Luis Cereijo

Roberto Valiente

Valeria Cuenca

Diana Juanita Mora

**BC3 Basque Centre for Climate**

**Change**

Manuel Franco

**University of Oslo**

Nanna Lien

Soren Holm

Sondre H Herstad

Biljana Meshkovska

**University of Oulu**

Sylvain Sebert

Rozenn Nedelec

Jouko Miettunen

Fahmida Sarker

Khin Hlaing (Yuyu)

Subhechchha Bhandari

Susanna Pätsi

**World Obesity Federation**

Angie Jackson-Morris

Matt O' Flynn

**BETTER4U** (Preventing obesity through biologically and behaviorally tailored interventions)

**Harokopio University of Athens**

George Dedoussis

Yannis Manios

Christos Diou

Panagiotis Moulos

Ioanna Panagiota Kalafati

Maria Kafyra

Panagiotis Symianakis

Eva Karaglani

Vasiliki Vavouraki

Christina Patmiou

Paris Kantaras

Panagiotis Alimisis

Anastasios Papamanolis

**Centro de Estudos e Investigacão em Dinâmicas Sociais e Saúde**

Ana Rito

Marta Gaspar

Fátima Martins

Matilde Vicente

Raquel Henriques

**Inserm**

Julie-Anne Nazare

Louise Seconda

Anestis Dougkas

**Region Hovedstaden**

Klaus Bønnelykke

Rebecca Kofod Vinding

David Horner

**European Food Information Council (EUFIC)**

Stephan Kampshoff

Darya Silchenko

**International Hellenic University**

Maria Hassapidou

Ioannis Pagkalos

Elena Patra

**Karolinska Institutet**

Ioannis Ioakeimidis

Anna Ek

Alkyoni Glympi

**Medical University of Vienna**

Eva Schernhammer

Magdalena Zebrowska

**University of Cyprus**

Constantinos Deltas

Stavros Gravas

Panagiota Veloudi

Alexis Kyriacou

Apostolos Malatras

**University of Helsinki**

Jaakko Kaprio

Teemu Palviainen

Gabin Drouard

Karri Silventoinen

Alvaro Obeso

**University of Navarra; CIBERobn**

Maira Bes-Rastrollo

Carmen Sayon-Orea

Miguel A. Martinez-Gonzalez

Cristina Razquin

Vanessa Bullon-Vela

**Ghent University**

Delfien Gryspeerdt

Nick Verhaeghe

Ruben Willems

Lieven Annemans

**SWPS University**

Aleksandra Luszczynska

Paulina Krzywicka

Zofia Szczuka

Hanna Zaleskiewicz

Anna Kornafel

**University of Tartu**

Anders Eriksson

**German Research Center for**

**Environmental Health**

Elisabeth Thiering

Marie Standl

**IUF - Leibniz Research Institute for Environmental Medicine**

Tamara Schikowski

Department of Environmental Immunology, Helmholtz Centre for Environmental Research – UFZ

Gunda Herberth

**VU Amsterdam**

Dorret I. Boomsma

René Pool

Suzanne Bruins

**University of Vienna**

Rodessa May Marquez

Olga Startseva

Nikolaus Forgó

**Weizmann Institute of**

**Science**

Eran Segal

Adina Weinberger

**Wings ICT Solutions Information & Communication Technologies S.A**

Vera Stavroulaki

Vangelis Argoudelis

Panagiotis Demestichas

Danai Malti

Gianna Karanasiou

Dimitris Plakas

Nikos Sintoris

**University of Cagliari**

Vassilios Fanos

Angelica Dessì

Luigi Atzori

Cristina Piras

Antonio Noto

Patrizia Baire

Matteo Mauri

Karolina Krystyna Kopeć

**BIOCLINICA SA**

Cristinel Gheorghiu

**Aristotle University of Thessaloniki**

Anastasios Delopoulos

Ioannis Sarafis

Alexandros Papadopoulos

Chrysa Episkopou

Dimitrios Aletras

**Tampere University Hospital and Tampere University**

Terho Lehtimäki

Nina Mononen

Pashupati P Mishra

Binisha H Mishra

Leo-Pekka Lyytikäinen

**University of Bern**

Stavroula Mougiakakou

Ioannis Papathanail

Rooholla Poursoleymani

Lubnaa Abdur Rahman

**QIMR Berghofer Medical Research Institute**

Nick Martin

Scott Gordon

**Bradford Institute for Health Research**

Gillian Santorelli

Ellena Badrick

John Wright

Amy Hough

**William Harvey Research Institute, Queen Mary University of London**

Eirini Marouli

**PAS GRAS** (promoting health throughout the life course to prevent obesity)

**University of Coimbra**

Alain Massart

Ana Teixeira

Anabela Marisa Azul

André Lázaro

Andreia Amaro

Andreia Silva

António Cruz

Aristides Machado-Rodrigues

Bruna Moreira

Bruno Manadas

Cláudia Pereira

Cristina Padez

Carina Magalhães

Carminda Morais

Catarina Parente

Célia Cabral

Daniela Rodrigues

Daniela Rosendo-Silva

Diana Sousa

Eduardo Lopes

Ermelindo Leal

Eugénia Carvalho

Flávio Reis

Francisco Pereira

Heloísa Gerardo

John Jones

Jorge Abrantes

José Teixeira

João Ramalho-Santos

Luís Grilo

Luís Rama

Maria Teresa Cruz

Miguel Castelo-Branco

Nuno Lourenço

Nuno Madeira

Paul Hardman

Paulo Matafome

Paulo Oliveira

Pedro Ferreira

Pedro Santos

Pelin Alpay

Rui Tavares

Sara Amaral

Sónia Pinho

Teresa Cunha-Oliveira

Vilma Sardão

**Università degli Studi di Bari Aldo Moro**

Agostino Di Ciaula

Danika Schepis

Harshitha Shanmugam

Mohamad Khalil

Piero Portincasa

**Uppsala Universitet**

Fleur Hukema

Jan Eriksson

Maria Pereira

Rutger Laterveer

Susanne Hetty

**Universidade Nova de Lisboa**

Joana Sacramento

Maria Paula Macedo

Rita Oliveira

Rita Patarrão

Sílvia Conde

Tatiana Burrinha

**Fundació Eurecat**

Antoni Caimari

Dorota Komar

Joan Teichenné

Núria Canela

Lorena Calderón-Pérez

Salvador Fernández-Arroyo

**Consiglio Nazionale delle Ricerche**

Amalia Gastaldelli

Egeria Scoditti

Liliana Cori

Silvia Sabatini

**Instituto Politécnico de Viana do Castelo**

Carla Faria

Isabel Araújo

Patrícia Vieira

Pedro Faria

Rita Pinheiro

Rui Pimenta

**Instituto Politécnico de Coimbra**

João Lima

Margarida Liz

Marlene Lages

Paulo Matafome

**Technische Universität München**

Adrian Jauch

Adriana Fontes

Hans Zischka

Judith Sailer

**Instytut Biologii Doświadczalnej im. M. Nenckiego PAN (Nencki Institute)**

Barbara Pakula

Magdalena Lebiedzinska-Arciszewska Agnieszka Dobrzyń

Justyna Janikiewicz

Grzegorz Sumara

**Mediagnost Gesellschaft für Forschung und Entwicklung GmbH**

Andrea Normann

Markus Langkamp

**Martin-Luther-Universität Halle-Wittenberg**

Marlene Rechtsteiner

Susann Weihrauch-Blüher

Susanne Kröber

**King’s College London**

Aakruti Kaikini

Afshan Malik

Dawei Chen

**Unidade Local de Saúde de Coimbra**

Cátia Barra

Helena Rodrigues

Lelita Santos

Patrícia Afonso-Mendes

**Instituto Pedro Nunes**

Ana Realinho

João Quintas

**Associação de Ginástica do Centro**

Ana Cláudia Guedes

**Associação Protetora dos Diabéticos de** **Portugal**

Carlos Penha Gonçalves

João Filipe Raposo

Laura Herrera

Maria Paula Macedo

Polina Dobroslavska

**European Society for Clinical Investigation**

Gemma Vilahur

**CoDiet** (Combatting diet related non-communicable disease through enhanced surveillance)

**AZTI**

Itziar Tueros

Sara Arranz

Mercedes Caro

Ainara Cano

Javier Amézaga

Gerard Marrugat

Laura García

Josean Montoya

Elena Santacruz

Amaia Barrena

Ainhoa Ruano

**Imperial College London**

Gary Frost

Benny Lo

Franco Sassi

Joram Posma

Robert Shorten

Isabel Garcia Perez

George Mylonas

Jack Olney

Monica Hill

Aygul Dagbasi

Baichen Lu

Donghee Choi

Pietro Ferraro

Jingmin Zhu

Adrian Rubio

Po-Wen Lo

Marek Rei

Ivana Balkan

Alexandra P. Halbish Rayner

**Czech Technical University**

Jakub Marecek

Xiaoyu He

Petr Rysavy

Ales Wodecki

Pavel Rytir

**Teagasc**

Orla O’Sullivan

Paul Cotter

Liam Walsh

**BIOMIC_AUTh**

Dimitrios Zaikis

Olga Begou

Olga Deda

Danai Rossiou

Dominiki Gallou

Alexandra Tiganouria

Ifigeneia Rizopoulou

Helen Gika

Georgios Theodoridis

**Ciber**

Josep A. Tur

Helmut Schröder

Cristina Bouzas

**CIC bioGUNE**

Oscar Millet

Nieves Embade

Rubén Gil

Ricardo Conde

**Microcaya**

Natalia Zaldua

Sabin Linaza

Ines Barretxeguren

Eduardo Aguaviva

Anabel Martinez

Oscar Carrancio

**Sciensano**

Stefanie Vandevijvere

**Tervise Arengu Instituut**

Marit Priinits

Diva Eeensoo

**The National and Kapodistrian University of Athens**

Gunopulos Dimitrios

Kalogeraki Vasiliki

**University of Nottingham**

**University of Trento**

Nicola Segata

Gloria Fackelmann

Federica Pinto

**University of Valencia**

Dolores Corella

Carolina Ortega-Azorin

José Vicente Sorlí

Olga Portolés

Oscar Coltell

**Bruker**

Birk Schütz

Iris Mangelschots

Claire Cannet

Hartmut Schäfer

**The Technion-Israel Institute of Technology**

Shie Mannor

Mark Kozdoba

**Istituto Superiore di Sanita**

Marco Silano

Anna Ceccarelli

Valentina De Cosmi

**HealthyW8** (Empowering Healthy Lifestyle Behaviour)

**Luxembourg Institute of Health**

Torsten Bohn

Mahesh Desai

Irina Carpusca

Manon Gantenbein

Alejandra Loyola Leyva

Laurent Malisoux

Jonathan Turner

Farhad Vahid

Michel Vaillant

Adriana Voicu

**Luxembourg Institute of Science and Technology**

Yannick Naudet

Christoph Stahl

**NIUM**

Alberto Norhona

Jorge Ribeiro

Adam Selamnia

Fernando Veloso

**German Research Centre for Artificial Intelligence**

Serge Autexier

Jan Janssen

**Virtech OOD**

Roumen Nikolov

Mariya Zheleva

Alexandre Chikalanov

Ventsislav Nikolov

Mariya Zheleva

**Leibniz Institute for Prevention Research and Epidemiology**

Sarah Forberger

Cassandra Omane

Laura Mewes

**Center for Agro-food Economics and Development**

Zein Kallas

Amelia Sarroca

Djamel Rahmani

**University of Gastronomic Sciences**

Serena Rinaldi

Maria Giovanna Onorati

Sara Casartelli

Andrea Devecchi

**Consiglio Nazionale delle Ricerche**

Arianna D`Ulizia

Alessia D`Andrea

**Agrifood Research and Technology Centre of Aragon**

Tiziana De Magistris

Cristina Gora

Jihan Halimi

**University of Évora**

Elsa Sousa De Lamy

Maria Perez Jimenez

Armando Raimundo

Nuno Batalha

Fernando Capela e Silva

Sofia Tavares

**Foundation Balearic Islands Health Research Institute**

Josep A. Tur

Cristina Bouzas

Marina Rodenas Munar

Silvia Garcia

Elena Ferragut Roig

**IRCCS Azienda Ospedaliero Universitaria di Bologna**

Giuseppe Tarantino

Lucia Brodosi

Maria Cristina Morelli

Michele Stecchi

**Technical University of Denmark**

Rikke Andersen

Gitte Ravn-Haren

Aleksandra Davydova

**University of Twente, Enschede**

Ying Wang

Ramon Que

**University of Coimbra**

Daniela Rodrigues

Helena Nogueira

Licinio Manco

Maria Raquel Silva

Monica Truninger

André Seabra

**Regional Cluster "North-East"**

Yoanna Ivanova

Boyko Doychinov

**Eindhoven University of Technology**

Pieter Van Gorp

Suzan Evers

Olga Glazunova

Charly Bastiaansen

Astrid Kemperman

**MEDEA SRL**

Pietro Dionisio

Francesco Agnoloni

Patrizia Zitelli

**Association Euro Atlantic Diplomacy Society**

George-Mihael Manea

**The European Nutrition for Health Alliance**

Joost Wesseling

Konstantina Maria Togka

**KNEIA S.L. SP**

Cristina Barragan Yebra

Christina Barragan Mesa

Ciro Avolio

**The European Federation of the Associations of Dietitians**

Marianna Kalliostra

Ezgi Kolay

Katarzyna Janiszewska

**Shift2Health** (Development and evaluation of nutritional strategies to reduce and prevent obesity in shift workers)

**University of Vienna**

Karl-Heinz Wagner

Katrin Scionti

Vanessa Schoissengeier

Ivana Vaclavkova

Leah Lund

Kilian Gandolf

Fabio Pfaehler

**FH Joanneum, Austria**

Anna Lena Aufschnaiter

Bianca Fuchs-Neuhold

Daniela Grach

Anika Kronberger

Monika Riederer

Marlies Wallner

Christina Höfler

Miriam Ressler

Simon Berner

Valentin Kraus

**Stichting Wageningen Research**

Monique Vingerhoeds

Logan Stuck

Meeke Ummels

Boshuizen, Hendriek

Maartje van den Belt

**Wageningen University**

Edith Feskens

Marco Mensink

Emiilie de Zoete

Jeanne Lagerweij

Desiree Lucassen

**University of Bremen**

Jilani Hannah

Urte Klink

Gerhardus Ansgar

**TTZ Bremenhaven**

Imke Matullat

Marie Shrestha

Anneli Rost

Ingo Klarholz

Benjamin Küther

**ENWHP**

Giuseppe Masanotti

Steve Bell

Richard Wynne

Karnaki Pania

Maria-Dolores Sole

**Medical University of Lodz**

Karolina Czarnecka

Monika Kolska

Jaroslaw Rakoczy

Magdalena Wrzesinska

Katarzyna Binder-Olibrowsk

Magdalena Wieczorkowska

Kinga Zel-Hans

Natalia Plociennik-Korycka

**Medical University of Vienna**

Kyriaki Papantoniou

Eva Schernhammer

Maria Wakolbinger

Eva Winzer

Sandra Haider

Isabel Santonja

Christine Ellersdorfer

**University of Copenhagen**

Wender Bredie

Helene Reinbach

Annemarie Olsen

Aikaterina Vasileiou

Catalina Cuparencu

Lars Dragsted

**The Akkermansia Company**

Willem de Vos

Peter Suenaert

Ivo Van Delft

Anneleen Segers

Helene Devroye

**KU Leuven**

Inge Depoortere

Alice Denis

Guillaume Vanotti

**Charité- Medical University of Berlin**

Achim Kramer

Bert Maier

**Erasmus University Medical Center**

Heidi Lammers-van der Holst

Coen Dros

Sanne Roove

**BioStreams** (Addressing childhood obesity in Europe)

**ICCS**

Dimitrios Koutsouris

George Matsopoulos

Eleftheria Vellidou

Ioannis Kakkos

Athanasios Anastasiou

Stavros Milioulis

Andreas Vezakis

Vassilis Apostolakos

Evika Karamaggioli

Eleni Chatzi

**Harokopio University of Athens**

Alexandra Karahaliou

Athanasia Kyrkili

Eirini Bathrellou

Georgios Saltaouras

Giannis Arnaoutis

Ismini Grapsa

Mary Giannakoulia

Meropi Kontogianni

Vasiliki Bountziouka

Eleni Politi

Ioannis Vondikakis

Dimitris Gkoulis

Yioula Lekka

George Dimitrakopoulos

**National and Kapodistrian University of Athens**

Penio Kassari

Vassiliki Papageorgiou-Anagnostou

Valerios Chatzianastasiou

Sofia-Maria Genitsaridi

Eleni Ramouzi

Marina Papadopoulou

Christos Nikitas

Evangelia Charmandari

**AINIGMA Techogies**

Marianna Panagiotidou

Athanasios Kakasis

**Vilabs**

Vassiliki Moumtzi

Danai Kyrkou

**I2Grow Srl**

Matteo Colombo

Sofia Silvola

Umberto Restrelli

**Tecreando**

Ioannis Vezakis

**Novelcore**

Nikos Alimpertis

Dimitrios Tsakalidis

**Blocks Health and Social Care EOOD**

Svetlin Hansov

Radka Savova

**University of Ioannina**

Dimitrios Fotiadis

Eleni Georga

Orestis Papagiannopoulos

Evgenia Lampropoulou

**Karolinska Institutet**

Billy Langlet

Ioannis Ioakeimidis

Alkyoni Glympi

**Computer Solutions Cyprus Ltd**

Stavros Pitoglou

Thelma Androutsou

Marilena Tarousi

John Filippas

**Vall d'Hebron University Hospital**

Andreea Ciudin-Mihai

Marta Comes Martinez

Eduard Mogas Vinals

Christina Aguilar Riera

Graciela Gastelum Varela

Ana Marcela Zapata Castellon

Jose Raul Herance Camacho

**Danish Committee for Health Education**

Andreas Jespersen

Ena Nielsen

**Martel Innovate**

Giada Martello

**European School Heads Association**

Eszter Salamon

Petra Van Haren

**University Medical Centrer Maribor**

Martin Bigec

Sergej Černčič

**University of Maribor**

Izidor Mlakar

Urska Smrke

**Obelisk** (Preventing childhood obesity to stay healthy throughout life)

**Inserm**

Amëlie Bonnefond

Philippe Froguel

Ralf Jockers

Julie Dam

**Beta**

Jayne Evans

Rebecca Markilie

Jo Boulding

**German Institue of Human Nutrition Potsdam-Rehbruecke (DlfE)**

Annette Schürmann

Meriem Ouni

Heike Vogel

Miriam Ulz

**Hospital del Mar Medical Research Institute**

Jana Selent

**Inserm Transfert**

Juliane Halftermeyer

**Lille University Hospital**

Philippe Froguel

Frédéric Gottrand

Florence Flamein

**Lund University**

Charlotte Ling

Alice Maguolo

Karl Bacos

**Nencki Institute of Experimental Biology**

Grzegorz Sumara

Dominika Malińska

**Public Hospitals of Paris**

Christine Poitou

**SIB Swiss Institute of Bioinformatics**

Mark Ibberson

Florence Mehl

Frédéric Burdet

Anne Niknejad

Iulian Dragan

Robin Liechti

Van Du Thoung Tran

Lou Götz

**Technical University of Munich**

Stuart McLennan

Marthe Smedinga

**The European Childhood Obesity Group Belgium (ECOG)**

Luigi Petito

Leila Mathy

David Thivel

**Tor Vergata University of Rome**

Vincenzo Atella

Andrea Piano Mortari

Pietro Monti

Francesca Marazzi

**University of Messina**

Malgorzata Gabriela Wasniewska

Tommaso Aversa

Giorgia Pepe

Domenico Corica

Debora Porri

Letteria Morabito

**University of Oulu**

Sylvain Sebert

Justiina Ronkainen

Rozenn Nedelec

Mavis Fosuaa Boateng

**University of Verona**

Anita Morandi

Frediana Tummino

Stephane Lobbens

Noemi Rita Colacione

**eprObes** (Preventing lifetime obesity by early risk-factor identification, prognosis and intervention)

**Consorcio CIBER**

Manuel Tena-Sempere

Concepcion Aguilera

Alexia Barroso

**Premium Research**

Javier Carrero

Eudald Casals Mercadal

Guadalupe González

**Eberhard Karls Universitaet Tuebingen UT**

Katrin Giel

Stephan Zipfel

Annica Doersam

Astrid Günther

Florian Junne

Isabelle Mack

Norbert Schmitz

Jana Throm

Katrin Ziser

**Universidad de Córdoba**

Manuel Tena-Sempere

Rafael Pineda

Juan Roa

Miguel Angel Sanchez-Garrido

María Jesús Vázquez

**University of Liege**

Anne-Simone Parent

Julie Fudvoye

Chloé Glachet

Charlotte Jacquinet

Quentin Terwagne

Anneline Pinson

**FIBICO – Fundación Investigación**

Biomédica de Córdoba

María Mercedes Gil-Campos

Katherine Flores-Rojas

María José de la Torre Aguilar

Francisco Jesús Llorente-Cantarero

Belén Pastor-Villaescusa

**Vilniaus** **Universiteto Ligonine**

**Santaros Klinikos Vulsk**

Augustina Jankauskiene

Zana Antonova

Karolis Azukaitis

Robertas Kemezys

Kajus Merkevicius

Justė Parnarauskienė

Ieva Jura Paulaviciene

Ausrine Pliauckiene

**Region Hovestaden - REGIONH**

Anders Juul

Nadia Micali

Stine Agergaard Holmboe

Elena Jansen

Kristian Almstrup

Anna Brieva-Toloza

Charlotte Ehlers Thomsen

Trine Koch Hueg

Cathrine Winding

**Fundación Investigación Hospital**

**General Universitario de Valencia**

Empar Lurbe

Julio Álvarez Pitti

**Uniwersytet Rzeszowski**

Artur Mazur

Natalia Dąbek

Aneta Radaczyńska

Justyna Wyszyńska

Edyta Łuszczki

**Consejo Superior Investigaciones Científicas (CSIC)**

Mario Fernandez Fraga

Juan José Alba

Agustin Fernandez

Raul Fernandez

Jennifer Kefauver

Virginia Lopez

Javier Menéndez

Annalisa Roberti

Pablo Santamarina

Juan Ramon Tejedor

Rocio Urdinguio

**Fundació Institut d’investigació**

**Biomèdica de Bellvitge**

Fernando Fernádez-Aranda

Susana Jiménez

Lucía Camacho

Lucero Munguia

**Amaris/ Mantulab**

Petar Atanasov

Yassine Talas

Alex Bravo Serrano

Adrianna Tryskuc

Thibaut Galba

Shanti Neff-Baro

**Uniwersytet Jagiellonski**

Dorota Drożdż

Mirosław Bik-Multaowski

Agnieszka Kozioł-Kozakowska

Przemko Kwinta

Małgorzata Wójcik

**Institut National de la Sante Et de la Recherche Medicale INSERM**Sebastien Bouret

Pierre-Yves Barelle

Amine Belfoul

Konstantina Chachlaki

Sreekala Nampoothiri

Vincent Prevot

Marialetizia Rastelli

Alicia Sicardi

**INCLIVIA**

Felip Vilella

**Turkish Society of Hypertension**

Serap Erdine

Ömer Faruk Beser

Haluk Cezmi Çokuğraş

Fügen Çullu Çokuğraş

**Universidad de Valencia**

Carlos Simón

Javier Gonzalez

Ana Ochando

1. One organization and four OBEClust members requested not to be affiliated with this position paper. [↑](#endnote-ref-1)
