# Supplementary material for: The case for primary prevention of obesity in the era of GLP-1 therapies
Source: Lancet Reg Health Eur. 2026 Apr 16;66:101679. doi: 10.1016/j.lanepe.2026.101679 (PMC13330260; doi:10.1016/j.lanepe.2026.101679)
Supplement: Collaborator list [file mmc2.docx]

**Collaborator list**

| **First name** | **Surname** |
| --- | --- |
| Aakruti | Kaikini |
| Achim | Kramer |
| Adam | Selamnia |
| Adina | Weinberger |
| Adrian | Jauch |
| Adrian | Rubio |
| Adriana | Fontes |
| Adriana | Voicu |
| Adrianna | Tryskuc |
| Afshan | Malik |
| Agnieszka | Dobrzyń |
| Agnieszka | Kozioł-Kozakowska |
| Agostino | Di Ciaula |
| Agustin | Fernandez |
| Aikaterina | Vasileiou |
| Ainara | Cano |
| Ainhoa | Ruano |
| Alain | Massart |
| Alberto | Norhona |
| Alejandra Loyola | Leyva |
| Aleksandra | Davydova |
| Aleksandra | Luszczynska |
| Ales | Wodecki |
| Alessia | D`Andrea |
| Alex | Bravo Serrano |
| Alexandra | Karahaliou |
| Alexandra | Tiganouria |
| Alexandra | Halbish Rayner |
| Alexandre | Chikalanov |
| Alexandros | Papadopoulos |
| Alexia | Barroso |
| Alexis | Kyriacou |
| Alfred | Wagtendonk |
| Alice | Denis |
| Alice | Maguolo |
| Alicia | Sicardi |
| Alkyoni | Glympi |
| Alvaro | Obeso |
| Amaia | Barrena |
| Amalia | Gastaldelli |
| Amelia | Sarroca |
| Amëlie | Bonnefond |
| Amine | Belfoul |
| Amy | Hough |
| Ana Cláudia | Guedes |
| Ana Marcela | Zapata Castellon |
| Ana | Ochando |
| Ana | Realinho |
| Ana | Rito |
| Ana | Teixeira |
| Anabel | Martinez |
| Anabela Marisa | Azul |
| Anastasios | Delopoulos |
| Anastasios | Papamanolis |
| Anders | Eriksson |
| Anders | Juul |
| André | Lázaro |
| André | Seabra |
| Andrea | Devecchi |
| Andrea | Normann |
| Andrea | Piano Mortari |
| Andreas | Jespersen |
| Andreas | Vezakis |
| Andreea | Ciudin-Mihai |
| Andreia | Amaro |
| Andreia | Silva |
| Anestis | Dougkas |
| Aneta | Radaczyńska |
| Angelica | Dessì |
| Angie | Jackson-Morris |
| Anika | Kronberger |
| Anita | Morandi |
| Anna | Banik |
| Anna | Brieva-Toloza |
| Anna | Ceccarelli |
| Anna | Ek |
| Anna | Kornafel |
| Anna Lena | Aufschnaiter |
| Annalisa | Roberti |
| Anne-Simone | Parent |
| Anne | Niknejad |
| Anneleen | Segers |
| Anneli | Rost |
| Anneline | Pinson |
| Annemarie | Olsen |
| Annette | Schürmann |
| Annica | Doersam |
| Antoni | Caimari |
| António | Cruz |
| Antonio | Noto |
| Apostolos | Malatras |
| Arianna | D`Ulizia |
| Aristides | Machado-Rodrigues |
| Armando | Raimundo |
| Artur | Mazur |
| Astrid | Günther |
| Astrid | Kemperman |
| Athanasia | Kyrkili |
| Athanasios | Anastasiou |
| Athanasios | Kakasis |
| Augustina | Jankauskiene |
| Ausrine | Pliauckiene |
| Aygul | Dagbasi |
| Baichen | Lu |
| Barbara | Pakula |
| Belén | Pastor-Villaescusa |
| Benjamin | Küther |
| Benny | Lo |
| Bert | Maier |
| Bianca | Fuchs-Neuhold |
| Biljana | Meshkovska |
| Billy | Langlet |
| Binisha H | Mishra |
| Birk | Schütz |
| Boshuizen | Hendriek |
| Boyko | Doychinov |
| Bruna | Moreira |
| Bruno | Manadas |
| Carina | Magalhães |
| Carla | Faria |
| Carlos | Penha Gonçalves |
| Carlos | Simón |
| Carmen | Sayon-Orea |
| Carminda | Morais |
| Carolina | Ortega-Azorin |
| Cassandra | Omane |
| Catalina | Cuparencu |
| Catarina | Parente |
| Cathrine | Winding |
| Cátia | Barra |
| Célia | Cabral |
| Charlotte | Ehlers Thomsen |
| Charlotte | Jacquinet |
| Charlotte | Ling |
| Charly | Bastiaansen |
| Chloé | Glachet |
| Christina | Aguilar Riera |
| Christina | Barragan Mesa |
| Christina | Höfler |
| Christina | Patmiou |
| Christine | Ellersdorfer |
| Christine | Poitou |
| Christoph | Stahl |
| Christos | Diou |
| Christos | Nikitas |
| Chrysa | Episkopou |
| Ciro | Avolio |
| Claire | Cannet |
| Cláudia | Pereira |
| Coen | Dros |
| Concepcion | Aguilera |
| Constantinos | Deltas |
| Cristina | Barragan Yebra |
| Cristina | Bouzas |
| Cristina | Gora |
| Cristina | Padez |
| Cristina | Piras |
| Cristina | Razquin |
| Cristinel | Gheorghiu |
| Danai | Kyrkou |
| Danai | Malti |
| Danai | Rossiou |
| Daniela | Grach |
| Daniela | Rodrigues |
| Daniela | Rosendo-Silva |
| Danika | Schepis |
| Darya | Silchenko |
| David | Horner |
| David | Thivel |
| Dawei | Chen |
| Debora | Porri |
| Delfien | Gryspeerdt |
| Desiree | Lucassen |
| Diana Juanita | Mora |
| Diana | Sousa |
| Dimitrios | Aletras |
| Dimitrios | Fotiadis |
| Dimitrios | Koutsouris |
| Dimitrios | Tsakalidis |
| Dimitrios | Zaikis |
| Dimitris | Gkoulis |
| Dimitris | Plakas |
| Diva | Eeensoo |
| Djamel | Rahmani |
| Dolores | Corella |
| Domenico | Corica |
| Dominika | Malińska |
| Dominiki | Gallou |
| Donghee | Choi |
| Dorota | Drożdż |
| Dorota | Komar |
| Dorret I. | Boomsma |
| Edith | Feskens |
| Eduard | Mogas Vinals |
| Eduardo | Aguaviva |
| Eduardo | Lopes |
| Edyta | Łuszczki |
| Egeria | Scoditti |
| Eirini | Bathrellou |
| Eirini | Marouli |
| Eleftheria | Vellidou |
| Elena | Ferragut Roig |
| Elena | Jansen |
| Elena | Patra |
| Elena | Santacruz |
| Eleni | Chatzi |
| Eleni | Georga |
| Eleni | Politi |
| Eleni | Ramouzi |
| Elisabeth | Thiering |
| Ellena | Badrick |
| Elsa Sousa | De Lamy |
| Emiilie | de Zoete |
| Empar | Lurbe |
| Ena | Nielsen |
| Eran | Segal |
| Erica | van den Akker |
| Ermelindo | Leal |
| Eszter | Salamon |
| Eudald | Casals Mercadal |
| Eugénia | Carvalho |
| Eva | Karaglani |
| Eva | Schernhammer |
| Eva | Schernhammer |
| Eva | Winzer |
| Evangelia | Charmandari |
| Evgenia | Lampropoulou |
| Evika | Karamaggioli |
| Ezgi | Kolay |
| Fabio | Pfaehler |
| Fahmida | Sarker |
| Farhad | Vahid |
| Fátima | Martins |
| Federica | Pinto |
| Felip | Vilella |
| Fernando | Capela e Silva |
| Fernando | Fernádez-Aranda |
| Fernando | Veloso |
| Flávio | Reis |
| Fleur | Hukema |
| Florence | Flamein |
| Florence | Mehl |
| Florian | Junne |
| Francesca | Marazzi |
| Francesco | Agnoloni |
| Francisco | Jesús Llorente-Cantarero |
| Francisco | Pereira |
| Franco | Sassi |
| Frédéric | Burdet |
| Frédéric | Gottrand |
| Frediana | Tummino |
| Fügen | Çullu Çokuğraş |
| Gabin | Drouard |
| Gary | Frost |
| Gemma | Vilahur |
| George-Mihael | Manea |
| George | Dedoussis |
| George | Dimitrakopoulos |
| George | Matsopoulos |
| George | Mylonas |
| Georgios | Saltaouras |
| Georgios | Theodoridis |
| Gerard | Marrugat |
| Gerhardus | Ansgar |
| Giada | Martello |
| Gianna | Karanasiou |
| Giannis | Arnaoutis |
| Gillian | Santorelli |
| Giorgia | Pepe |
| Gitte | Ravn-Haren |
| Giuseppe | Masanotti |
| Giuseppe | Tarantino |
| Gloria | Fackelmann |
| Graciela | Gastelum Varela |
| Grzegorz | Sumara |
| Guadalupe | González |
| Guillaume | Vanotti |
| Gunda | Herberth |
| Gunopulos | Dimitrios |
| Haluk | Cezmi Çokuğraş |
| Hanna | Zaleskiewicz |
| Hans | Zischka |
| Harshitha | Shanmugam |
| Hartmut | Schäfer |
| Heidi | Lammers-van der Holst |
| Heike | Vogel |
| Helen | Gika |
| Helena | Nogueira |
| Helena | Rodrigues |
| Helene | Devroye |
| Helene | Reinbach |
| Helmut | Schröder |
| Heloísa | Gerardo |
| Hoang-Ha | Nguyen |
| Ieva | Jura Paulaviciene |
| Ifigeneia | Rizopoulou |
| Imke | Matullat |
| Ines | Barretxeguren |
| Inge | Depoortere |
| Ingo | Klarholz |
| Ioanna | Panagiota Kalafati |
| Ioannis | Ioakeimidis |
| Ioannis | Kakkos |
| Ioannis | Pagkalos |
| Ioannis | Papathanail |
| Ioannis | Sarafis |
| Ioannis | Vezakis |
| Ioannis | Vondikakis |
| Irina | Carpusca |
| Iris | Mangelschots |
| Isabel | Araújo |
| Isabel | Garcia Perez |
| Isabel | Santonja |
| Isabelle | Mack |
| Ismini | Grapsa |
| Itziar | Tueros |
| Iulian | Dragan |
| Ivana | Balkan |
| Ivana | Vaclavkova |
| Ivo | van Delft |
| Izidor | Mlakar |
| Jaakko | Kaprio |
| Jack | Olney |
| Jakob | Tarp |
| Jakub | Marecek |
| Jan | Eriksson |
| Jan | Janssen |
| Jana | Selent |
| Jana | Throm |
| Jaroslaw | Rakoczy |
| Javier | Amézaga |
| Javier | Carrero |
| Javier | Gonzalez |
| Javier | Menéndez |
| Jayne | Evans |
| Jeanne | Lagerweij |
| Jennifer | Kefauver |
| Jeroen | Lakerveld |
| Jet | van de Geest |
| Jihan | Halimi |
| Jilani | Hannah |
| Jingmin | Zhu |
| Jo | Boulding |
| Joan | Teichenné |
| Joana | Sacramento |
| Joanneum | Austria |
| João Filipe | Raposo |
| João | Lima |
| João | Ramalho-Santos |
| João | Quintas |
| John | Filippas |
| John | Jones |
| John | Wright |
| Joline | Beulens |
| Jonathan | Turner |
| Joost | Wesseling |
| Joram | Posma |
| Joreintje | Mackenbach |
| Jorge | Abrantes |
| Jorge | Ribeiro |
| Jorrit | van Uhm |
| Jose Raul | Herance Camacho |
| José | Teixeira |
| José Vicente | Sorlí |
| Josean | Montoya |
| Josep A. | Tur |
| Josine | Stuber |
| Jouko | Miettunen |
| Juan José | Alba |
| Juan | Ramon Tejedor |
| Juan | Roa |
| Judith | Sailer |
| Julia | Díez |
| Juliane | Halftermeyer |
| Julie-Anne | Nazare |
| Julie | Dam |
| Julie | Fudvoye |
| Julio | Álvarez Pitti |
| Justė | Parnarauskienė |
| Justiina | Ronkainen |
| Justyna | Janikiewicz |
| Justyna | Wyszyńska |
| Kajus | Merkevicius |
| Kalogeraki | Vasiliki |
| Kamille | Almer Bernsdorf Torp |
| Karl-Heinz | Wagner |
| Karl | Bacos |
| Karnaki | Pania |
| Karolina Krystyna | Kopeć |
| Karolina | Czarnecka |
| Karolis | Azukaitis |
| Karri | Silventoinen |
| Katarzyna | Binder-Olibrowsk |
| Katarzyna | Janiszewska |
| Katherine | Flores-Rojas |
| Katrin | Giel |
| Katrin | Ziser |
| Khin | Hlaing |
| Kilian | Gandolf |
| Kinga | Zel-Hans |
| Kirsten | Schroll Bjørnsbo |
| Klaus | Bønnelykke |
| Konstantina Maria | Togka |
| Konstantina | Chachlaki |
| Kristian | Almstrup |
| Kyriaki | Papantoniou |
| Lars | Dragsted |
| Laura | García |
| Laura | Herrera |
| Laura | Mewes |
| Laurent | Malisoux |
| Leah | Lund |
| Leila | Mathy |
| Lelita | Santos |
| Leo-Pekka | Lyytikäinen |
| Letteria | Morabito |
| Liam | Walsh |
| Licinio | Manco |
| Liesbeth | van Rossum |
| Lieven | Annemans |
| Liliana | Cori |
| Logan | Stuck |
| Lorena Calderón | Pérez |
| Lou | Götz |
| Louise | Seconda |
| Lubnaa | Abdur Rahman |
| Lucero | Munguia |
| Lucia | Brodosi |
| Lucía | Camacho |
| Lucrezia | Bertoni |
| Luigi | Atzori |
| Luigi | Petito |
| Luis | Cereijo |
| Luís | Grilo |
| Luís | Rama |
| Maartje | van den Belt |
| Magdalena | Lebiedzinska-Arciszewska |
| Magdalena | Wieczorkowska |
| Magdalena | Wrzesinska |
| Magdalena | Zebrowska |
| Mahesh | Desai |
| Maira | Bes-Rastrollo |
| Malgorzata | Gabriela Wasniewska |
| Małgorzata | Wójcik |
| Manon | Gantenbein |
| Manuel | Franco |
| Manuel | Tena-Sempere |
| Marco | Mensink |
| Marco | Silano |
| Marcus | Langkamp |
| Marek | Rei |
| Margarida | Liz |
| Maria Cristina | Morelli |
| Maria Giovanna | Onorati |
| María José | de la Torre Aguilar |
| Maria Paula | Macedo |
| Maria Raquel | Silva |
| Maria Teresa | Cruz |
| Maria-Dolores | Sole |
| Maria | Hassapidou |
| María | Jesús Vázquez |
| Maria | Kafyra |
| María Mercedes | Gil-Campos |
| Maria | Pereira |
| Maria | Perez Jimenez |
| Maria | Siwa |
| Maria | Wakolbinger |
| Marialetizia | Rastelli |
| Marianna | Kalliostra |
| Marianna | Panagiotidou |
| Marie | Shrestha |
| Marie | Standl |
| Marilena | Tarousi |
| Marina | Papadopoulou |
| Marina | Rodenas Munar |
| Mario | Fernandez Fraga |
| Marit | Priinits |
| Mariusz | Wieckowski |
| Mariya | Zheleva |
| Mark | Ibberson |
| Mark | Kozdoba |
| Marlene | Lages |
| Marlene | Rechtsteiner |
| Marlies | Wallner |
| Marta | Comes Martinez |
| Marta | Gaspar |
| Marthe | Smedinga |
| Martin | Bigec |
| Mary | Giannakoulia |
| Matilde | Vicente |
| Matt | O' Flynn |
| Matteo | Colombo |
| Matteo | Mauri |
| Mavis | Fosuaa Boateng |
| Meeke | Ummels |
| Mercedes | Caro |
| Meriem | Ouni |
| Meropi | Kontogianni |
| Michel | Vaillant |
| Michele | Stecchi |
| Miguel A. | Martinez-Gonzalez |
| Miguel Adriano | Sanchez-Lastra |
| Miguel Angel | Sanchez-Garrido |
| Miguel | Castelo-Branco |
| Miriam | Ressler |
| Miriam | Ulz |
| Mirosław | Bik-Multaowski |
| Mohamad | Khalil |
| Mohan | Raju |
| Monica | Hill |
| Monica | Truninger |
| Monika | Kolska |
| Monika | Riederer |
| Monique | Vingerhoeds |
| Nadia | Micali |
| Nanna | Lien |
| Natalia | Dąbek |
| Natalia | Paduszyńska |
| Natalia | Plociennik-Korycka |
| Natalia | Zaldua |
| Nick | Martin |
| Nick | Verhaeghe |
| Nicola | Segata |
| Nieves | Embade |
| Nikolaus | Forgó |
| Nikos | Alimpertis |
| Nikos | Sintoris |
| Nina | Mononen |
| Nishit | Patel |
| Noemi Rita | Colacione |
| Norbert | Schmitz |
| Nuno | Batalha |
| Nuno | Lourenço |
| Nuno | Madeira |
| Núria | Canela |
| Olga | Begou |
| Olga | Deda |
| Olga | Glazunova |
| Olga | Portolés |
| Olga | Startseva |
| Ömer | Faruk Beser |
| Orestis | Papagiannopoulos |
| Orla | O’Sullivan |
| Oscar | Carrancio |
| Oscar | Coltell |
| Oscar | Millet |
| Pablo | Santamarina |
| Panagiota | Veloudi |
| Panagiotis | Alimisis |
| Panagiotis | Demestichas |
| Panagiotis | Moulos |
| Panagiotis | Symianakis |
| Paris | Kantaras |
| Pashupati P | Mishra |
| Patrícia | Afonso-Mendes |
| Patrícia | Jakubek-Olszewska |
| Patrícia | Vieira |
| Patrizia | Baire |
| Patrizia | Zitelli |
| Paul | Cotter |
| Paul | Hardman |
| Paulina | Krzywicka |
| Paulo | Matafome |
| Paulo | Oliveira |
| Pavel | Rytir |
| Pedro | Faria |
| Pedro | Ferreira |
| Pedro | Santos |
| Pelin | Alpay |
| Penio | Kassari |
| Petar | Atanasov |
| Peter | Suenaert |
| Petr | Rysavy |
| Petra | Van Haren |
| Philippe | Froguel |
| Piero | Portincasa |
| Pierre-Yves | Barelle |
| Pieter | van Gorp |
| Pietro | Dionisio |
| Pietro | Ferraro |
| Pietro | Monti |
| Polina | Dobroslavska |
| Po-Wen | Lo |
| Przemko | Kwinta |
| Quentin | Terwagne |
| Radka | Savova |
| Rafael | Pineda |
| Ralf | Jockers |
| Ramon | Que |
| Raquel | Henriques |
| Raul | Fernandez |
| Rebecca | Kofod Vinding |
| Rebecca | Markilie |
| Renate | Meeusen |
| René | Pool |
| Ricardo | Conde |
| Richard | Wynne |
| Rikke | Andersen |
| Rita | Oliveira |
| Rita | Patarrão |
| Rita | Pinheiro |
| Robert | Shorten |
| Robertas | Kemezys |
| Roberto | Valiente |
| Robin | Liechti |
| Rocio | Urdinguio |
| Rodessa May | Marquez |
| Rooholla | Poursoleymani |
| Rosalie | Bakker |
| Roumen | Nikolov |
| Rozenn | Nedelec |
| Rubén | Gil |
| Ruben | Willems |
| Rui | Pimenta |
| Rui | Tavares |
| Rutger | Laterveer |
| Sabin | Linaza |
| Salvador | Fernández-Arroyo |
| Sandra | Haider |
| Sanne | Roove |
| Sara | Amaral |
| Sara | Arranz |
| Sara | Casartelli |
| Sarah | Forberger |
| Scott | Gordon |
| Sebastien | Bouret |
| Serap | Erdine |
| Serena | Rinaldi |
| Serge | Autexier |
| Sergej | Černčič |
| Shanti | Neff-Baro |
| Shie | Mannor |
| Sílvia | Conde |
| Silvia | Garcia |
| Silvia | Sabatini |
| Simon | Berner |
| Sofia-Maria | Genitsaridi |
| Sofia | Silvola |
| Sofia | Tavares |
| Sondre H | Herstad |
| Sónia | Pinho |
| Soren | Holm |
| Sreekala | Nampoothiri |
| Stavros | Gravas |
| Stavros | Milioulis |
| Stavros | Pitoglou |
| Stavroula | Mougiakakou |
| Stefanie | Vandevijvere |
| Stephan | Kampshoff |
| Stephan | Zipfel |
| Stephane | Lobbens |
| Steve | Bell |
| Stine | Agergaard Holmboe |
| Stuart | McLennan |
| Subhechchha | Bhandari |
| Susana | Jiménez |
| Susann | Weihrauch-Blüher |
| Susanna | Pätsi |
| Susanne | Hetty |
| Susanne | Kröber |
| Suzan | Evers |
| Suzanne | Bruins |
| Svetlin | Hansov |
| Sylvain | Sebert |
| Tamara | Schikowski |
| Tatiana | Burrinha |
| Teemu | Palviainen |
| Teresa | Cunha-Oliveira |
| Terho | Lehtimäki |
| Thao | Minh Lam |
| Thelma | Androutsou |
| Thibaut | Galba |
| Tina | Termansen |
| Tiziana | De Magistris |
| Tommaso | Aversa |
| Torsten | Bohn |
| Trine | Koch Hueg |
| Ulf | Ekelund |
| Ulla | Toft |
| Umberto | Restrelli |
| Urska | Smrke |
| Urte | Klink |
| Valentin | Kraus |
| Valentina | De Cosmi |
| Valeria | Cuenca |
| Valerios | Chatzianastasiou |
| Van | Du Thoung Tran |
| Vanessa | Bullon-Vela |
| Vanessa | Schoissengeier |
| Vangelis | Argoudelis |
| Vasiliki | Bountziouka |
| Vasiliki | Vavouraki |
| Vassiliki | Moumtzi |
| Vassiliki | Papageorgiou-Anagnostou |
| Vassilios | Fanos |
| Vassilis | Apostolakos |
| Ventsislav | Nikolov |
| Vera | Stavroulaki |
| Vilma | Sardão |
| Vincent | Prevot |
| Vincenzo | Atella |
| Virginia | Lopez |
| Wender | Bredie |
| Willem | de Vos |
| Xiaoyu | He |
| Yannick | Naudet |
| Yannis | Manios |
| Yassine | Talas |
| Ying | Wang |
| Yioula | Lekka |
| Yoanna | Ivanova |
| Zana | Antonova |
| Zein | Kallas |
| Zofia | Szczuka |
